# Evaluating artifact‐free four‐dimensional computer tomography with 16 cm detector array

**DOI:** 10.1002/acm2.70056

**Published:** 2025-02-24

**Authors:** Inhwan Yeo, Wei Nie, Jiajin Fan, Mindy Joo, Michael Correa, Qianyi Xu

**Affiliations:** ^1^ Department of Advanced Radiation and Proton Therapy INOVA Schar Cancer Institute Fairfax Virginia USA; ^2^ Radiation Oncology Thomas Jefferson University Philadelphia Pennsylvania USA

**Keywords:** 16 cm long detector, 4DCT, artifacts, respiration irregularity

## Abstract

**Purpose:**

To evaluate a 16 cm‐array axial four‐dimensional computer tomography (4DCT) in comparison with a 4 cm‐array 4DCT in the presence of respiration irregularity.

**Method:**

Ten traces of lung tumor motion from CyberKnife treatments were imported to move the lung cylinder, containing a spherical target, of a phantom. Images were acquired for the lung that moved to each of the 10‐positions/phases (1) step‐wisely by nominal helical scan at each movement (ground truth), (2) continuously by 4D scan with the 16 cm array, and (3) the same with the 4 cm array, involving table shift. Irregularities, consisting of baseline shift and/or amplitude change of the traces in their second periods, affected #3 scan only in its second table position. The full‐widths at half maximum of the target in the direction of the motion were determined on the average (Ave) CT, maximum‐intensity (Mip) CT, and a phase (MP) CT that is associated with the maximum error, comparing #2 and 3 with #1. Three tumor‐shaped targets were also imaged, and overlap ratios of them from #2 and 3 with the targets from #1 were inter‐compared. Hounsfield unit (HU)s of the targets were also compared.

**Results:**

The average difference in the spherical‐target length between #2 and #1 was found to be 0.28 ± 0.15 cm on AveCT, 0.00 ± 0.18 cm on MipCT, and 0.07 ± 0.06 cm on MPCT, showing agreement. The average difference between #3 and #1 was 0.34 ± 0.23 cm on AveCT, 0.48 ± 0.31 cm on MipCT, and 0.56 ± 0.50 cm on MPCT, showing disagreement. The overlap ratios were better with #2 than with #3 for all tumor‐shaped targets in each phase CT and MipCT, but they were not perfect for #2 due to motion averaging and phase sorting limitations. The differences in HUs were smaller with #2 than with #3, but not fully satisfactory with #2.

**Conclusion:**

4DCT with the 16 cm array needs to be used to minimize the impact of the irregularity.

## 1 INTRODUCTION

Accurate delineation of tumors with respiratory motion through quantification of their motion is essential for their successful treatment. The motion can be significant, as up to 5 cm of displacement was reported for tumors that are located in the lung and liver.^1^ Efforts to account for the motion have been made to acquire and delineate tumor contours in their individual respiratory phase in four‐dimensional CT (4DCT). The concept of 4DCT was first introduced by Taguchi after the fourth temporal dimension was added to the conventional three‐dimensional (3D) cone‐beam computer tomography (CBCT).^2^ This was followed by extensive studies on 4DCT by using fan beams.^3^, ^4^, ^5^, ^6^

Four‐dimensional CT starts from continuous scanning of a patient in either helical or axial modes while recording concurrently a respiratory signal from a surrogate that is placed externally on the patient. Each scanned slice has a temporal stamp, representing a unique respiratory phase of the signal. Subsequently, the scanned slices are respectively sorted into different respiratory phases. In routine practice, ten phases are typically adopted. Instead of phases, amplitudes of the surrogate motion may be used for the sorting.

It is known that for 4DCT the sorting method based on the surrogate inevitably introduced motion artifacts due to imperfect correlation between internal tumor motion and external signals^7^ and irregular respiratory motion patterns.^8^, ^9^, ^10^ The artifacts could be contributed by the irregularity when a limited length of CT detector arrays is used. Employed by most centers, they cannot encompass the scan length, required by clinical needs, necessitating multiple positions of table on which a patient is placed, and ten to twenty respiratory cycles. During the lengthy period, the patient respiratory pattern could change. This causes some respiratory phases to be incorrectly sampled and, even if they are correctly sampled, respiratory traces are not correlated between neighboring table positions, creating artifacts in the reconstructed 4DCT.

In recent years, the concept of volumetric 4DCT was brought up to alleviate the motion artifacts in 4DCT, especially in either diaphragm or liver region.^11^ The volumetric 4DCT was typically acquired on high‐end CTs with 256 detector arrays for the GE revolution scanner (General Electric, Boston, MA) or with 320 arrays for the Toshiba Aquilion One scanner (Canon, Otawara, Japan). The detector arrays could cover a scan range of 16 cm (with slice resolution of 0.625 or 0.5 mm), which would be sufficient to encompass and scan the majority of moving tumors in one respiratory cycle without requiring table shift. This would remove the motion artifacts mentioned above due to the limited width of CT detector arrays. Also, the time of one rotation of the scanner could be done within 0.28 and 0.35 seconds on the two scanners, respectively.^12^ This would reduce motion blurring and improve temporal resolution.

Reports on the volumetric 4DCT have been sparce due to its high price and limited availability. Coolens et al. evaluated the 4DCT with the 320 arrays that helically scanned spherical and morphological‐tumor targets, while they were moving sinusoidally and realistically following respiratory traces.^11^ They showed advantage of the 4DCT over a shorter‐length 4DCT. In this study, we aimed to evaluate the 4DCT that are scanned axially using the 256 arrays when respiration irregularities are present (artifacts upon table shift). While the group used the programmed traces and the known target volumes as ground truth information, we used nominal helical 3DCT images of the targets that are static at the time of scanning as ground truths. Also, our evaluation was based on the imaged target size or volume for not only each phase, but also averaged and integrated phases which are clinically used (i.e., their overlap ratios with the ground truth values). We evaluated the 4DCT in Hounsfield Units (HUs) as well.

## 2 METHODS

### 2.1 Phantom and patient selection

**FIGURE 1:**
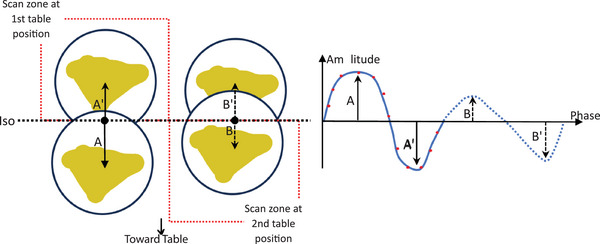
Phantom setup and motion driven by a respiratory trace.

A phantom (Quasar, IBA, Inc) with a lung‐mimicking insert was used for this study. The insert contained a cavity that can be filled with a target. Above the phantom, a platform was placed, that moved vertically, simulating abdomen motion, as the insert moved superior‐inferiorly in the direction of gantry and table, simulating respiratory lung motion. The lung motion was driven by each of ten respiratory traces of superior‐inferior tumor motion that were collected from CyberKnife treatments in our center, based on a motion model that is benchmarked by stereo imaging of tumor. In more detail, for each trace among entire periods that spanned approximately 30 min, a portion of two or more respiratory cycles (periods) were chosen which contained an irregularity of a baseline shift and/or an amplitude change that are present in patients’ respiration. Figure 1 illustrates the motion of the cavity containing a target, driven by a selected trace in which the second portion (i.e., second period) showed an amplitude decrease. The phantom was setup by aligning the center of the cavity at its base position to the isocenter of the GE CT unit that utilizes a minimum 4 cm and a maximum 16 cm as the lengths of detector array that is aligned in the superior‐inferior direction. The cavity moved inferiorly when the trace moved toward the peaks (maximum inhale) from the position of zero amplitude (base position of the cavity), reaching the amplitudes of A or B; it moved superiorly when the trace moved toward the valleys (maximum exhale), reaching the amplitude of A′ and B′.

### 2.2 Image acquisition

Imaging in this study included three methods. (1) The CT was run to image a spherical target filling the cavity that was moved step‐wisely to each of the ten‐positions/ten‐phases of the first period by fast helical scan at each position. This served as the ground‐truth (GT) image for each phase in this study because the scan has been nominally used clinically and usage of the static target at the time of scanning did not involve motion artifacts. (2) The CT was run with the target moving continuously by axial 4D scan with a 16 cm array (4D_16_). The two scans did not involve table shift. (3) CT was similarly run using the entire two‐periods portion of the trace that moved the target continuously by axial 4D scan with a 4 cm array involving one table shift (4D_4_).

When the 16 cm length was utilized, at one table position of the target, the entire cavity was scanned axially throughout a period of motion (i.e., the first period in Figure 1 in this study). When the 4 cm length was utilized, the length covered only a superior portion of the target that was placed in its first table position during the first period (see the superior scan zone) and moved with amplitudes of A and A′. An inferior portion of the target was scanned (see the inferior scan zone), after the target was shifted to a second table position and moved with B and B′ differently characterizing the two respiratory limits, respectively. Therefore, the maximum extents of the motion captured by the 4 cm length was characterized by the amplitudes of A′ and B in superior and inferior limits, respectively (baseline shift if there was did not cause the change of the superior limit from A′ to B′). Note that at each table position, CT scanning covered only a portion of the moving target driven by an associated portion of trace (first or second period), that may come into the assigned scan zone. In this system of CT scanning, if the assigned portion of trace changed its shape between two different table positions, the whole target image could not physically represent the actual target shape at certain phases for which two table positions are needed to image the entire volume of the target. The irregularity, sampled from the ten patient treatments, was intended to affect the target image of the scan with the 4 cm length in its second table position, provided the adjoint border of the two table positions was aligned to the center of the target. For the GT and 4D_16_ scans, the first‐period portion of the trace only was utilized, thereby unaffected by the irregularity. Among the ten traces, one had the baseline shift; five had the amplitude change; four had both. The respiratory periods of the patient traces varied between 4 and 11 s, while the rotation time of the CT bore varied between 0.28 and 1 s. The time was chosen to be as small as possible to minimize the motion during one imaging rotation. While the GT scan did not involve the phantom movement during imaging rotation of the CT, the 4D_16_ and 4D_4_ did, thereby involving the motion averaging. The image slice thickness was 0.25 cm. The above process was repeated using three other tumor targets, irregularly‐shaped, and small enough to be placed in the cavity, which was created by printing the gross tumor volumes (GTVs) in patient plans with a 3D printer (AXIOM20, Airwolf, NV) and their associated traces, respectively.

This experimental setup was designed to visualize the impact of the artifacts in the image of the target in a manner that was readily geometrically interpretable. When tumor‐shaped targets were inserted in the spherical cavity, due to their asymmetric shapes they could not be centrally positioned, so that their centers were not aligned to the 4 cm length edge for the scan of 4D_4_. The interpretation of their phase images needs understanding of their off‐centric placements; nevertheless, their motion extents from the imaging isocenter (not the target center). In real life, the diseased targets, whether centrally or not, may or may not be aligned to the edges; instead, other organs such as diaphragm or lung may be aligned to the edges, so that the artifacts may be created in them if aligned to the edges. Constrained by their non‐rigid natures also, their geometric shapes may be interpreted.

### 2.3 Analysis

The GT scan, gathered from all phases, were processed to generate the maximum‐intensity CT (MipCT) and the average‐intensity CT (AveCT) in Velocity (Varian Medical Systems, Palo Alto, CA). The 4D_16_ and 4D_4_ scans were processed in Advantage Workstation (GE Health Care, Chicago, IL) to provide phase images, MipCT, and AveCT. The three sets of images from 4D_16_ and 4D_4_ were respectively compared with those of GT. For the comparison, the full‐widths at half maximum (FWHM) of the HU profiles across the spherical targets in the direction of the motion were determined in AveCT, MipCT, and a phase image that was associated with the maximum error among all phase images. For the tumor targets that are irregularly shaped, the overlap ratios of the volumes of them in each phase image and MipCT of 4D_16_ and 4D_4_ to those of GT were respectively determined and evaluated. The average HUs were also determined and compared for the spherical and actual targets that were contoured in phase images and Ave images of GT, 4D_16_ and 4D_4_. In this paper, the targets shown in each phase image and AveCT are named as gross tumor volume (GTV), the target in MipCT as integrated GTV (iGTV), and the target with an added margin to iGTV as planning target volume (PTV).

## 3 RESULTS

**FIGURE 2:**
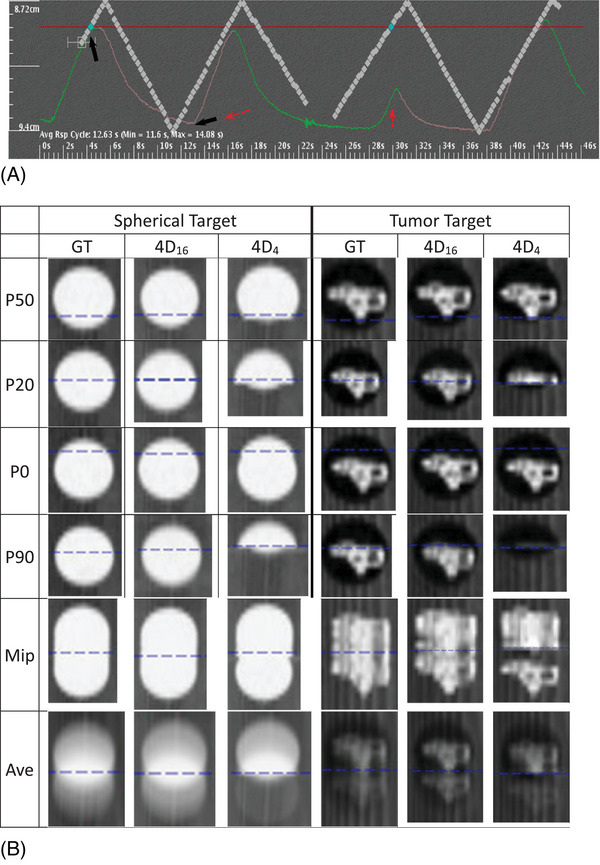
Patient I: Images acquired for one patient case with his/her respiratory trace for the spherical and tumor targets. (A) The trace used for image acquisition of GT, 4D_16_, and 4D_4_. (B) Acquired images at representative phases of 50, 20, 0, 90, Mip, and Ave of the three with the traces of (A). In (A), the first portion of the trace was utilized for GT as well as 4D_16_. The amplitudes at solid and dashed arrows determine the extent of target motion that are quantified in Table 1 for this patient. In (B), the blue horizontal line is at isocenter. The patient was #6 in Table 1.

Figure 2A shows a phantom motion, driven by a selected trace in this study, which was represented in Advantage Workstation by the movement of reflective markers of a motion tracking system (Varian medical system, Palo Alto, CA), that was placed on the platform. The movement of the markers showed the same trend to that of the actual trace with a reduced motion extent. The diamond symbols in the figure represent the positions of the traces used for phase sorting. They were at the interval of approximately 3%, based on the time interval of image reconstruction. The first group of the diamonds corresponds to the portion of the trace that was utilized at the first table position of image acquisition, covering two peaks of similar magnitudes of amplitudes; the second group corresponds to the portion at the second table position, covering two peaks of different magnitudes; the two groups were separated by a 1/2 s for a table travel, during which image acquisition was not performed. The second portion had a similar baseline at maximum exhale (at the valley of the diamonds); the peak‐to‐valley height of the amplitude (vertical axis of the trace) was reduced for the first peak (from 1.97 to 1.0 cm), but slightly increased for the second peak. The first group only was used for the scans of GT and 4D_16_ and the two groups were used for the scan of 4D_4_.

Overall and phase images of Figure 2: Figure 2B shows acquired images at representative phases of 50, 20, 0, 90, Mip, and Ave of the GT, 4D_16_, and 4D_4_ using the trace in Figure 2A for the spherical and tumor targets. The blue horizontal line represents the level at the isocenter. The 4D_4_ images of the spherical target showed image shortage in the direction of the motion at phases 50, 20, and 90, due to the assignment of each phase to the smaller trace of the second portion (the first peak), thereby not traveling the target as inferiorly as the first portion did for GT and 4D_16_. At phase 0 of 4D_4_ scan, unlike the other phases the target was slightly longer than that of GT in the region below the isocenter line due to the assignment of the phase to the greater peak of the second portion. The performance of 4D_16_ and 4D_4_ were quantified in Table 1 that provided (1) lengths of the spherical target in the direction of motion on MipCTs and AveCTs of GT, 4D_16_, and 4D_4_, respectively, and a phase CT of the maximum error of 4D_4_ and (2) HU of the target on AveCTs of the three scans. The table was fully evaluated later in this paper. The case of Figure 2 represents patient #6 in the table, whose tumor target size was 2.6 cm in its longest dimension and that after motion integration was 3.5 cm in the direction of motion. For phase 90, the phase of the maximum error of 4D_4_, the length for 4D_4_ showed the difference of 1.39 cm in “4D_4_‐GT”, while the length for 4D_16_ agreed with that of GT with the difference of 0.11 cm in “4D_16_‐GT”, demonstrating the reliability of 4D_16_. When tumor target I was used the shortage was visible at phases 20 and 90 only, differing from those for the spherical target, due to the asymmetric and irregular shape, and the off‐centric positioning of the target in the spherical cavity, relative to the motion extent and trend of the assigned portion of the trace. This was evaluated later incorporating the performance of 4D_16_ and 4D_4_ on all three tumor targets.

**TABLE 1:** Quantified error in the spherical target length in the direction of the motion at FWHM and HU of 4D_16_ and 4D_4_. Ten patient traces were used. ∼sorting difference was involved. *Absolute values were provided, because the second portion of the trace of Figure 2A was different from the first portion in amplitudes (greater/smaller) and we were interested in the magnitude of the difference. HU is average HU of iGTV contoured in AveCTs for the three CTs.

	Length (cm)																		HU		
	Mip							Ave					a phase						Ave		
pt	GT	4D_16_	4D_16_ ‐GT	4D_16__ trace‐GT	4D_4_	4D_4_ ‐GT	4D_4__ trace‐4D_4_	GT	4D_16_	4D_16_ ‐GT	4D_4_	4D_4_ ‐GT	GT	4D_16_	4D_16_‐GT	4D_4_	4D_4_ ‐GT	Phase	GT	4D_16_	4D_4_
1	5.06	5.01	−0.05	−0.06	5.70	0.64	−0.15	3.27	3.46	0.19	3.61	0.34	3.07	3.24	0.17	4.69	1.62	90	−323.2	−312.9	−339.8
2	4.18	4.11	−0.07	−0.08	4.87	0.69	0.13	3.21	3.41	0.20	3.73	0.52	3.07	3.15	0.08	3.35	0.28	20	−262.4	−260.2	−296.6
3	4.14	4.13	−0.01	0.01	3.45	−0.69	−0.10	3.22	3.45	0.23	2.96	−0.26	3.07	3.13	0.06	2.52	−0.55	20	−266.3	−271.4	−238.8
4	3.83	3.82	−0.01	−0.03	4.25	0.42	0.00	3.18	3.27	0.09	3.60	0.42	3.07	3.13	0.06	3.42	0.35	20	−239.3	−231.7	−235.6
5	4.36	4.34	−0.02	−0.17	4.03	−0.33	0.12	3.32	3.61	0.29	3.17	−0.15	3.08	3.11	0.03	3.04	−0.04	20	−292.2	−290.7	−288.6
6	5.13	4.94	−0.19	−0.16	5.20	0.07	−0.17	3.88	4.26	0.38	3.18	−0.70	3.11	3.22	0.11	1.72	−1.39	90	−348.4	−351.1	−309.0
7	4.58	4.46	−0.12	−0.05	4.56	−0.02	−0.01	3.34	3.74	0.40	3.39	0.05	3.11	3.15	0.04	2.96	−0.15	20	−289.7	−308.0	−282.6
8	4.92	4.87	−0.05	−0.19	4.57	−0.35	−0.15	3.66	3.98	0.32	3.72	0.06	3.13	3.19	0.06	2.69	−0.44	90	−326.7	−334.5	−307.6
9	4.24	4.23	−0.01	−0.20	3.77	−0.47	−0.17	3.46	3.58	0.12	3.26	−0.20	3.11	3.14	0.03	2.68	−0.43	10	−271.7	−271.1	−242.3
10	4.87	5.36∼	0.49	0.38	3.80	−1.07	−0.08	3.73	4.26∼	0.53	3.06	−0.67	3.16	3.14	−0.02	1.86	−1.30	80	−327.8	−372.8	−279.4
Ave	4.53	4.53	0.00	−0.06	4.42	0.48*	0.06	3.43	3.70	0.28	3.37	0.34*	3.10	3.17	0.07	2.99	0.56*		−294.8	−300.4	−282.0
Std	0.45	0.49	0.18	0.17	0.70	0.31	0.11	0.25	0.35	0.14	0.28	0.24	0.03	0.06	0.06	0.75	0.50		35.4	43.9	34.3

MipCT of Figure 2: The two targets in 4D_4_ respectively agreed with those in GT and 4D_16_ at the superior edges, based on the common amplitude (valley of the 1st period) that defines the limits of GT, 4D_16_, and 4D_4_. For the spherical target of 4D_4_, the problematic sorting of phase 90 (described in the preceding paragraph) did not affect the MipCT significantly, in part assisted by the sorting of phase 0 (described in the preceding paragraph), except for the localized, lateral constriction of the target just below the isocentric line. However, it affected the MipCT of the tumor target as shown by the gap (about 0.7 cm), just below the isocentric line, in the image of Figure 2B. While qualitatively shown in Figure 2B, for patient #6 the length of 4D_16_ (4.94 cm) was compared with that of GT for the spherical target, showing a difference of 0.19 cm in the value of “4D_16_‐GT” in Table 1. This difference came from the motion averaging of 4D_16_ in addition to experimental errors associated with this study (setup, motor accuracy driving motion, and phase sorting difference). The length of 4D_4_ (5.20 cm) was compared with that of GT, showing a small difference of 0.07 cm in the value of “4D_4_‐GT”, based on the explanation provided in the preceding paragraph. Due to the rigid nature of the motion and the target, the length of the MipCT could be calculated. Provided that the actual trace of Figure 2A that drove the motion had the peak amplitude situated 0.79 cm above the base of 0 cm (first arrow) and that of the valley 1.18 cm below the base (second arrow from left), the target had 1.97 cm as the full extent of travel; the diameter of the target was added, so the target length was calculated to be 4.97 cm (0.79 + 1.18 + 3.0), which agreed with the measured length of 5.13 cm of the target in MipCT of the GT for patient #6 in Table 1. The difference between them was provided as 0.16 cm in “4D_16__trace‐GT”, Table 1. The length of the target of the 4D_4_ could also be calculated by using two amplitude values: one is 1.18 cm at the valley of the first portion (first dashed arrow); the other is 0.85 cm at the smaller peak of the second portion (second dashed arrow); the length was calculated to be 5.03 cm which was different from the measured length by 0.17 cm, as provided in “4D_4__trace‐4D_4_”.

AveCT of Figure 2: As shown in Figure 2B, across the AveCTs and the MipCTs of the two targets for 4D_16_ and 4D_4_, HU values varied, and the variance differed visually from that of GT. This can be explained by the disagreement previously described in their phase images. The spherical target (GTV) size was determined differently between 4D_16_ and 4D_4_ from that of GT with the difference of 0.38 cm in “4D_16_‐GT” and 0.7 cm in “4D_4_‐GT”, shown in Table 1.

**FIGURE 3:**
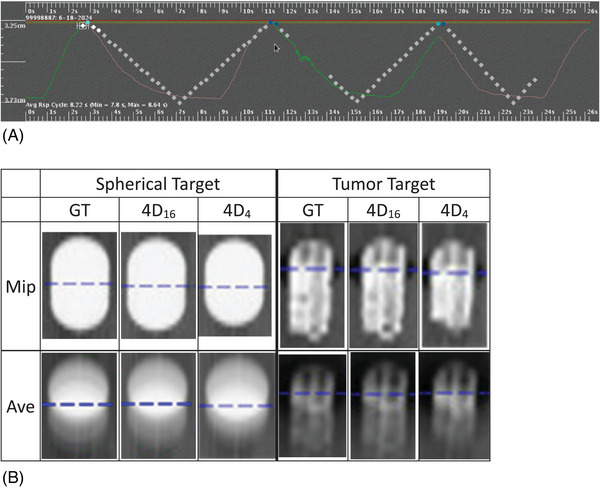
Patient II: Images acquired for another patient case with his/her respiratory trace for the spherical and tumor targets. (A) The trace used for image acquisition of GT, 4D_16_, and 4D_4_. (B) MipCT and AveCT of the three with the traces of (A). iGTV in 4D_4_ of tumor target II was 0.7 cm shorter than that of GT in the direction of motion. The patient is #8 in Table 1.

Figure 3 similarly shows the results of patient II. Figure 3A shows the two portions of the respiratory trace used for 4D_16_ and 4D_4_ with a slight baseline increase and an amplitude decrease of the second portion. Compared with patient I, the extents in ratio and magnitude of the amplitude decrease (from 1.73 to 1.3 cm) were not as great. For tumor target II, the shape and size were completely different; the longest dimension of the actual target was 1.8 cm and that of iGTV was 3.50 cm. Also, the second portion consisted of a single peak, while that of patient I consisted of two peaks of different magnitudes of amplitudes between them. Therefore, while the sorting for patient I utilized the two peaks which characterized phase images and MipCT, the sorting for patient II had to utilize the single peak solely with less impact of the irregularity in his/her images. Figure 3B shows the acquired images of Mip and Ave of GT, 4D_16_, and 4D_4_ with the traces of (A) for the spherical and actual targets. The change of the trace contributed to the visual shortage of iGTV on MipCT of 4D_4_ for both targets, compared with those of GT and 4D_16_ and similarly for GTV on AveCT of 4D_4_ for the tumor target. The finding for iGTV was quantified to be 0.35 cm in “4D_4_‐GT”, and was greater than that of patient I (0.07 cm) for the spherical target. The difference of “4D_4_‐GT” was found to be smaller than that of “4D_16_‐GT” (0.06 < 0.32) for GTV in AveCT, which was evaluated later in this paper. Besides the above findings, other findings were similar to those of the patient I, as quantified in Table 1.

**FIGURE 4:**
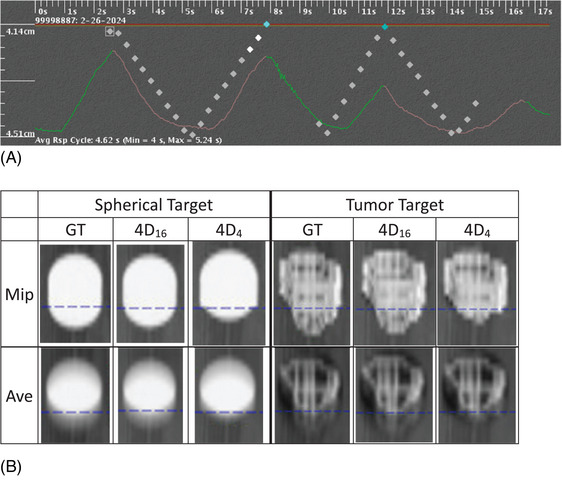
Patient III: Images acquired for another patient case with his/her respiratory trace and tumor target. (A) The trace used for image acquisition of GT, 4D_16_, and 4D_4_. (B) MipCT and AveCT of the three with the traces of (A). iGTV of 4D_4_ of tumor target III was 0.5 cm shorter than that of GT in the direction of motion. The patient is #9 in Table 1.

Figure 4 similarly shows the results of patient III. Figure 4A shows the two portions of the respiratory trace used for 4D_16_ and 4D_4_ with an amplitude decrease of the second portion. The relative extent of the amplitude decrease was greater (from 1.04 to 0.6 cm) than those of patients I and II. The tumor shape and size were different as well: the longest dimension of tumor was 2.8 cm and that of the iGTV was 3.8 cm. Figure 4B shows the acquired images of Mip and Ave of GT, 4D_16_, and 4D_4_ with the traces of (A) for the spherical and tumor targets. The change of the trace contributed to the shortage of iGTV on MipCT of 4D_4_ for both targets, compared with those of GT and 4D_16_, more visibly than the other two patient cases for the spherical target. This was due to the greatest reduction rate of the amplitudes, 0.6/1.04. Note that the disagreement of the length of iGTV of 4D_4_ with that of GT by 0.47 cm in “4D_4_‐GT” was greater than those of patients I (0.07 cm) and II (0.35 cm), as provided in Table 1 for patient #9.

**FIGURE 5:**
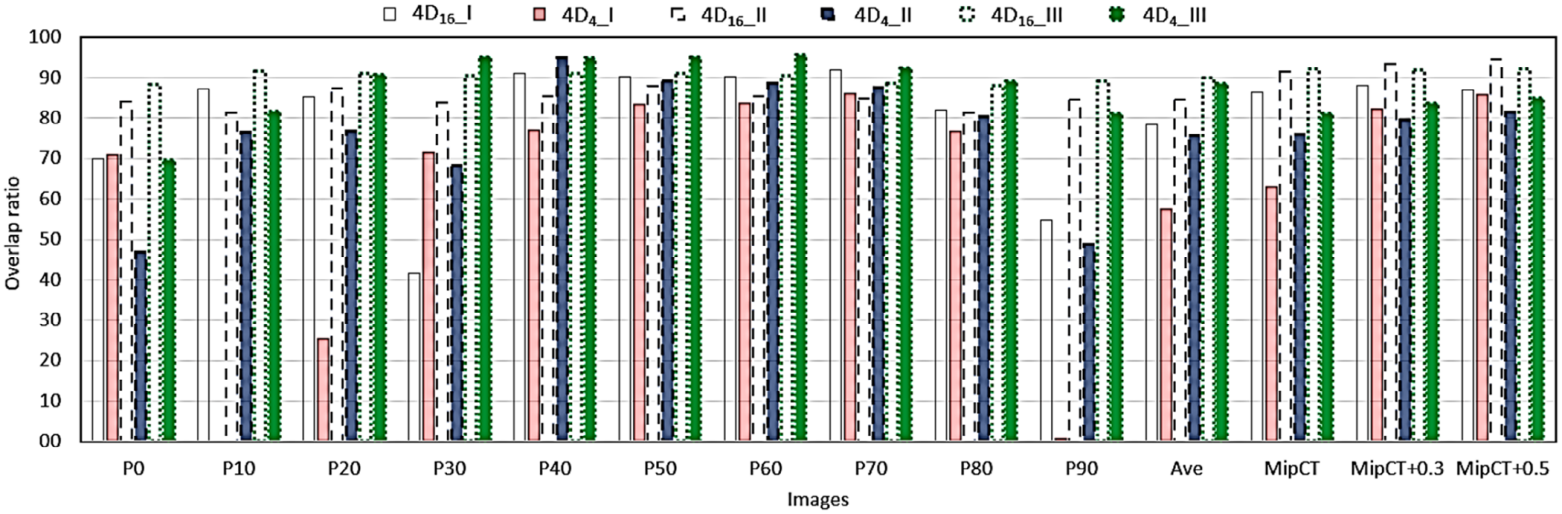
Overlap ratios of targets in phase images, MipCT, and MipCT with margins of 4D_16_ and 4D_4_ and the ratio averaged across all phases (Ave) to target in each comparable GT image. 16 cm_I and 4 cm_I stand for 4D_16_ and 4D_4_, respectively, of patient I. For each image, three sets of the ratio are presented for three patients, respectively, from left to right; each set included the ratios for 4D_16_ and 4D_4_.

Figure 5 shows the overlap ratios of each of the three actual targets in phase images (GTV), MipCT (iGTV), Mip + 0.3 (a margin of 0.3 cm added to iGTV), and Mip+0.5 to the target in each comparable image of GT. For each image, the ratios were provided for 4D_16_ and 4D_4_ and for the three targets (I–III) consecutiely from left to right. It showed varying agreement of the targets of 4D_16_ and 4D_4_ to that of GT as the phase changed, affected by the difference in the amplitude of each sorted phase compared to that of GT for the same phase. The overall performance of 4D_16_ for target I (4D_16__I) was shown by an average overlap ratio of 78.4% ± 16.6% across all phases with a minimum 41.5% at phase 30, affected by the motion averaging and the experimental errors (including contouring accuracy) in addition to the difference stated above. Note that the difference and the motion‐averaging effects are likely to be more significant in rapid‐gradient regions of the trace. The performance of 4D_4_ (4D_4__I) was found to be worse, as indicated by an average ratio of 57.5% ± 33.1% with its standard deviation value greater than that of 4D_16_ due to the irregularity included in its acquisition. The zero value of the ratio at phase 10 came from the off positioning of the target, while 0.6% at phase 90 came from the image miss with the target volume of <0.1 cm^3^ (visible in Figure 2B). The average GTV volumes imaged by GT, 4D_16_, and 4D_4_ were found to be 1.75 ± 0.03, 1.73 ± 0.05, and 1.30 ± 0.65 cm^3^, respectively, additionally demonstrating the impact of the irregularity on 4D_4_. The aforementioned irregularity can be in part explained by the length of GTV in the direction of the motion, determined to be approximately 1.3 cm, that is significantly smaller than the peak‐valley amplitude of 2.2 cm (length of iGTV of 4D_4_ scan (5.20 cm, patient 6 of Table 1)−target diameter (3 cm)). This indicates the relative significance of the motion extent to the GTV size, and therefore the impact of the irregularity. Similarly to the performance of GTVs in phase images, iGTV of 4D_16_ overlapped with that of GT better than that of 4D_4_ did (i.e., the gap), shown in the data for MipCT. The ratio was improved, approaching to the value for 4D_16_ when margins were added to iGTV for 4D_4_, incorporating the gap, but not as clearly for 4D_16_ due to the similarity between the volumes of 4D_16_ and GT. For target II, the average overlap ratio of 4D_16_ (4D_16__II) was better than that of 4D_4_ (4D_4__II), although not as dramatic as the betterment for target I. The minimum ratio for 4D_4_ was found to be 46.9% at phase 0, due to the most pronounced off‐positioning of GTV at the phase (second‐peak amplitude vs. first‐peak amplitude, Figure 3A), provided the volume was 1.46 cm^3^, a close value to the target volume of 1.44 ± 0.01 cm^3^. Note that the volumes of 4D_16_ and 4D_4_ were 1.44 ± 0.02 and 1.42 ± 0.11 cm^3^, respectively. The GTV length was approximately 1.3 cm, comparable to the amplitude of 1.57 cm, estimated for patient #8 for 4D_4_. This explains the average ratio of 76% that is significantly greater than 57.5% of target I. Similarily to the finding of the average ratio, for iGTV the overlap ratio was worse for 4D_4_. However, adding margins to iGTV for 4D_4_ did not improve the overlap ratio as much as they did for target I, as less irregularity impacted it for phase sorting, combined with the size and shape of the target and its relative positioning to the isocenter. For target III, differing from the findings for the other targets the average overlap ratio of 4D_4_ was similar to that of 4D_16_. At phase 0, the ratio for 4D_4_ was smallest with 69.6%, affected mostly by the smaller size of GTV, 4.89 cm^3^, compared with the GTV volume of GT, 6.42 ± 0.05 cm^3^. Note that the volumes were 6.44 ± 0.04 and 6.06 ± 0.52 cm^3^ for 4D_16_ and 4D_4_, respectively. The GTV length was 2.3 cm, significantly greater than the estimated amplitude of 0.77 cm for 4D_4_. This explains the average ratio of 88.6%, significantly greater than those for targets I and II. Therefore, the irregularity did not affect 4D_4_ as much. Similarly to the finding for target II, the ratio improved by only 3.6% as the margins were added to iGTV. The trend that the overlap ratios increased as the margins were added to iGTV similarly applies to the spherical target, because when margins were isotropically added to iGTV the difference in “4D_4_‐GT” stayed the same, although the overal length/volume of PTV was increased. Note that the overlap ratios of GTVs and iGTV of 4D_16_ to those of GT were not perfect, although 4D_16_ utilized one table position.

**FIGURE 6:**
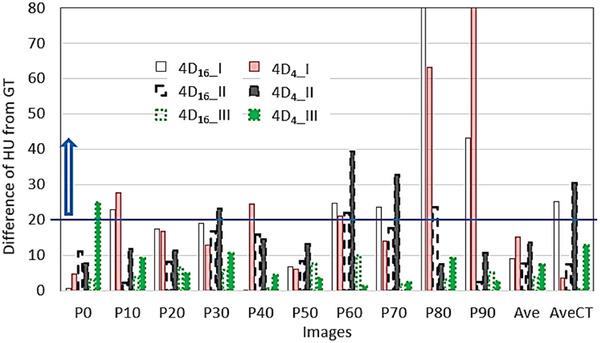
Differences in HU in targets that were contoured in phase images, the difference averaged across all phases (Ave), and the differences in average images (AveCT) of 4D_4_ and 4D_16_ from HU in target in each comparable GT image. 16 cm_II and 4 cm_II stand for 4D_16_ and 4D_4_, respectively, of patient II. For each image, three sets of the differences are presented for three patients, respectively, from left to right; each set included the differences for 4D_16_ and 4D_4_.

Figure 6 shows for the three targets the difference in the average HUs of GTV that was contoured in each phase image, the difference averaged across all phases, and the difference in the AveCTs of 4D_4_ and 4D_16_ from the HU of GTV in each comparable GT image. For target I, the HUs averaged across all phase images of GT, 4D_4_, and 4D_16_ were determined to be −359.8 ± 5.2, −375.0 ± 53.4, and −368.9 ± 30.7, respectively. The latter two exhibited differences smaller than 20 from the former, which is a recommended tolerance that can lead to a dose difference to 1%.^13^, ^14^ Due to the relatively large standard deviation values, the differences were not meaningful. Compared with the HU of each phase image of GT, at the images of phases 10, 40, 60, 80, and 90 of 4D_4_ the differences were found to be greater than 20 and smaller than 161, as shown in the figure (see arrow). The maximum difference of 161 was observed at phase 90 where the imaged target volume was smaller than 0.01 cm^3^. The same was found at phases 10, 20, 60, 80, and 90 of 4D_16_ with differences greater than 20 and smaller than 82. Although the average HUs did not violate the tolerance, the HUs of the above phase images did, contributed by the sorting of each phase of 4D_16_ and 4D_4_ that determined the volume of the target (nonuniform and irregularly shaped). Note that the 3D printing of the actual target did not provide a perfectly uniform target, which however modelled inhomogeneous nature of intratumor density distributions favorably for this study. When the target is uniform, such as the spherical target, a HU difference greater than 20 was still observed among GTVs in phase images where the volumes were erroneously reduced, so the portion of the target edge with different HUs than HUs in the interior targe regions was relatively increased. Therefore, this factor has additionally contributed to the HU difference for the tumor target. For target II, across GTVs of all phase images of GT, the average HU was −387.0 ± 3.6. The average HUs of 4D_16_ and 4D_4_ were −379.4 ± 11.0 and −373.4 ± 13.6, respectively, which were all close to the GT value within 20. At phases 60 and 80 of 4D_16_, HUs were different from the GT value by greater than 20 and smaller than 24; at phases 30, 60, and 70 of 4D_4_, HUs were different by greater than 20 and smaller than 40. For target III, the average HUs of 4D_16_ and 4D_4_ were −456.1 ± 3.7 and −452.6 ± 6.7, respectively, which were close to that of GT, −460.0 ± 2.5, within 20. Only at phase 0 of 4D_4_, the HU was different by 25.

The HU accuracy in AveCT is described in the followings. The difference in HU in GTV of the aveCT of 4D_4_ was about 40 from that of GT for the spherical target of Figure 2, as shown in patient #6, Table 1; the difference was 3.5 from that of GT (−670.2) for tumor target I (from patient #6), as shown in AveCT of Figure 6. The difference for the case of 4D_16_ was less than the tolerance for the spherical target, but it was 25.1, greater than the tolerance for tumor target I. The differences for both 4D_16_ and 4D_4_ were smaller than the tolerance for the spherical target of Figure 3, as shown in patient 8, Table 1, but it was greater than the tolerance for tumor target II for 4D_4_ only (−607.7 vs. −638.3), as shown in Figure 6. The difference for 4D_4_ only was greater than the tolerance for the spherical target, as shown in patient 9, Table 1, but those for both 4D_16_ and 4D_4_ were smaller than the tolerance for tumor target III of Figure 4, as shown in Figure 6. Overall, the average HU in GTV of AveCT tends to be with greater differences for 4D_4_.

MipCT of Table 1: The average length of iGTV of GT was measured to be 4.53 ± 0.45 cm with the maximum of 5.13 cm and the minimum of 3.83 cm, indicating various extents of motion used for this study, relative to 3 cm as the diameter of the spherical target. The length of iGTV in 4D_16_ was determined very close to that of GT with an average difference of 0.00 ± 0.18 cm in “4D_16_‐GT”. The difference of 4D_4_ from GT was found to be significant, as shown in “4D_4_‐GT” with an average of 0.48 ± 0.31 cm, due to the irregularity of the second portion of the traces used. The value of 0.48 is outside the 0.00 ± 1.96σ of “4D_16_‐GT”, where σ is 0.18, giving a confidence, greater than 95% under the Gaussian model, that 4D_4_ is inferior to 4D_16_ in its accuracy in providing iGTV. Note that it was found that the value of 0.49 cm in “4D_16_‐GT” for patient #10 was caused by the problematic phase sorting that was performed for the patient: out of two peaks included in the portion of trace used for GT and 4D_16_, the smaller peak was used for the phase 0 of GT, but the greater peak was used for that of 4D_16_, causing the length of iGTV extended more inferiorly (overall longer) for the latter. The sorting difference could be a part of clinical practice, so this datum was included in the average value of 4D_16_ across the ten patients. The average value of “4D_16__trace‐GT” was determined to be small, −0.06 ± 0.17 cm, as expected and explained (note: undesirable sorting was not involved in the generation of “4D_16__trace”). Similarly, 4D_4_ showed a small average value of −0.06 ± 0.11 cm for “4D_4__trace‐4D_4_”.

AveCT of Table 1: In AveCT, the GTV lengths of 4D_16_ and 4D_4_ showed average differences of 0.28 ± 0.14 cm (in “4D_16_‐GT”) and 0.34 ± 0.24  cm (in “4D_4_‐GT”), respectively. The determination of these values was affected by the gradual decrease of the target edge profile on AveCT, rather than a rapid drop of the profile on MipCT. Therefore, depending on the sampling point, they might differ. This also caused the determination susceptible to motion averaging and experimental errors on all phases, while the determination on MipCT was susceptible to those on the maximum inhale and exhale phases mostly. The two values of 4D_4_ and 4D_16_ were not significantly different from each other, based on the relative magnitude of the σ values to the associated average values, respectively. The average HU of GTV of 4D_4_ did not show a difference from that of GT by greater than the tolerance, although for five patients individually the differences were greater than 30 and smaller than 48.4 (maximum at patient #10).

Phase CT of Table 1: The maximum difference in “4D_4_‐GT” and its associated phase were found from various phases for each patient. The GTV length of 4D_4_ showed an average difference of 0.56 ± 0.5 cm in “4D_4_‐GT”. However, the length of 4D_16_ showed close agreement to the length of GT with an average difference of 0.07 ± 0.06 cm in “4D_16_‐GT”, confirming the accuracy of 4D_16_ for phase‐specific imaging. This finding from the phase images confirmed that the use of 4D_4_ cannot provide accurate dose in each phase anatomically. The maximum difference of “4D_16_‐GT” and that of “4D_4_‐GT” among the ten patient cases were found from patient #1, 0.17 and 1.62 cm, respectively. This was due to the position of the associated phase of 90 at a high gradient region of 0.5 cm travel per second of the associated trace, liable to a phase shift for sorting, and an increase of the peak amplitude of the second period by 0.7 cm for the case of 4D_4_.

## 4 DISCUSSIONS

The usage of the uniform‐density sphere with ten respoiratory traces had its limiation by not modeling the nonuniform density and irregular shapes of tumor, so the tumor targets were additionally used in this study. Through the usage of the two types of the targets, we found that their imaging suffers from geometrical inaccurcy (motion averaging and phase sorting issues included), due to the respiratory irregularity, that led to HU differences which are greater than 20. For photon planning, the difference in HU and those in the target locations and shapes of GTV from AveCT and iGTV from MipCT may be important in moving sites. For proton planning, robust planning is performed modeling uncertainties in patient (therefore, target) positioning and HU. Note that density override in the target is also done. Therefore, the impact of the geometrical inaccuracy of the target is mitigated, while the difference in HU does not need consideration. Whether the density is overriden or not and/or the robust optimization is done or not, the geometrical miss can be a serious issue, particularly in hypofractionated treatment, as the target can miss irradiation in a part of full phases, as demonstrated in this study. Unlike 4D_4_, phase images of 4D_16_ did not show significant image missing, although not perfect image matching (see overlapping ratio, Figure 5), which then is justified, and desired to be validated for 4D dose, compared with that with GT, dosimetrically quantifying the impact of the miss. To our knowledge, the geometrical accuracy only, not dose accuracy, was investigated by prior studies.^15^, ^16^

A study by Coolens et al.^11^ on the helical 4DCT with the 320‐detector array evaluated its temporal performance against programmed motions and known target volumes. This study evaluated the axial 4DCT with the 256 array differently against the fast helical scan of a static target. Some of further different approaches we took were as follows. This study evaluated in not only temporal phase CTs, but also AveCT and MipCT that are used clinically for a moving target. The overlap ratios of the targets, not just their volumes, when respiration irregularities were present allowed us to evaluate the geometrical accuracy of the two 4DCTs of 4D_16_ and 4D_4_. This study also evaluated the two in HUs, and demonstrated that both 4D_16_ and 4D_4_ were associated with inaccurate HUs beyond the tolerance of 20 that could have dosimetrical consequences.^13^, ^14^

Note that when the table shift is involved, oversampling using more than a period, phase resorting, and a selective use of the regular portion of a trace exhibited during 4DCT^17^ have been utilized to reduce the associated artifacts. With 4D_16_, the artifacts are intrinsically resolved by using one trace and one table position without requiring these efforts. This offers an option of repeat scanning over multiple respiratory periods at one table position, thereby incorporating respiratory irregularity in the definition of tumor, as demonstrated by Coolens et al.^11^ Currently, the vendor of the axial 4D_16_ scan does not allow 4D image sorting when a table shift is employed. Therefore, when the required scan length exceeds 16 cm, the acquired images need to be fused to a longer scan that can be a conventional helical scan. In this study, we did not investigate on the imaging doses of 4D_16_ and 4D_4_, as their difference was small (about 6%).

## 5 CONCLUSION

This study newly evaluated the accuracy of tumor delineation in 4DCT with axial 4D_16_, compared with GT, when various respiratory traces of patients’ tumors under treatment were employed, and quantified the geometrical accuracy of 4D_4_, including its phase CT, AveCT, and MipCT, in artifacts due to table shift when respiratory irregularities were involved after the shift, using a spherical target and three tumor‐shaped targets. The accuracy of 4D_16_ in phase‐specific target geometry and HUs was not perfect, and questionable in part when the tumor targets were employed, due to the effects of motion averaging and phase sorting associated with its image acquisition that were different from those of GT. The accuracy of 4D_4_ in HUs were worse than that of 4D_16_ for both targets, due to the respiratory irregularity. As a next step, this study suggests comparative clinical studies between the 16 cm scan and the limited‐length scan in relation to the motion artifacts. The 4D dose calculation, that verifies intended radiation therapy of moving targets, requires accurate phase images of the targets and neighboring organs in terms of geometrical shapes and HUs, that may be provided by 4D_16_. However, the accuracy of 4D_16_ for the calculation, against its geometrical accuracy limitation and HU inaccuracy (>20) in some of phase images, is a topic of further investigation. The quantified results of this study cautions the usage of the short‐length 4DCT when the irregularities are involved and/or when the traces show rapid gradients in amplitude/time, which can lead to undesirable phase sorting and/or artifacts due to table shift. The benefit demonstrated through this study per the cost of 4D_16_ needs to be evaluated considering the entire scope of motion management that includes tumor tracking, a respiration control, and/or ITV‐to‐PTV margins, provided that tumors may exhibit different motions than that at the time of image acquisition.

## AUTHOR CONTRIBUTIONS

Inhwan Yeo has contributed to research planning, experiments, evaluation, and manuscript writing. Weil Nie has contributed to research planning, experiments, evaluation, and manuscript writing. Jiajin Fan has contributed to research planning. Mindy Joo has contributed to manuscript writing. Michael Correa contributed to experiments and manuscript writing. Qianyi Xu has contributed to research planning, evaluation, and manuscript writing.

## CONFLICT OF INTEREST STATEMENT

The authors declare no conflicts of interest.
